# A novel biomimetic nanomedicine system with anti-inflammatory and anti-osteoporosis effects improves the therapy efficacy of steroid-resistant nephrotic syndrome

**DOI:** 10.1186/s12951-021-01165-z

**Published:** 2021-12-13

**Authors:** Jian Li, Mingyi Zhao, Xinying Xiang, Qingnan He, Rong Gui

**Affiliations:** 1 Department of Blood Transfusion, The Third Xiangya Hospital, Central South University, Changsha, 410013 China; 2 Department of Pediatrics, The Third Xiangya Hospital, Central South University, Changsha, 410013 China; 3 School of Life Sciences, Central South University, Changsha, 410013 China

**Keywords:** Steroid-resistant nephrotic syndrome, Biomimetic, Glycyrrhizic acid, Inflammatory

## Abstract

Clinically, steroid-resistant nephrotic syndrome (SRNS) is always prolonged and difficult to treat and easily develops into end-stage renal disease, resulting in a low survival rate. Strategies to reverse steroid resistance and reduce the long-term use of high doses of steroid medicines are urgently needed. In this study, a novel nanoparticle drug system (Pm-GCH) with a core–shell structure was designed. Metal–organic frameworks, synthesized by glycyrrhizic acid (G) and calcium ions (Ca^2+^) loaded with hydrocortisone (H) were the core of the nanoparticles. Platelet membrane vesicles were the shells. The natural platelet membrane endows Pm-GCH with good biocompatibility and the ability to promote immune escape. In addition, under the chemotaxis of inflammatory factors, platelet membranes assist Pm-GCH in nonspecific targeting of the inflammatory sites of the kidney. Under an inflammatory acid environment, GCH slowly degrades and releases glycyrrhizic acid and hydrocortisone. Glycyrrhizic acid inhibits the inactivation of hydrocortisone, jointly inhibits the activity of phospholipase A2 (PLA2) and the classic activation pathway of complement C2, blocks the production of inflammatory factors, plays an anti-inflammatory role, and enhances the efficacy of hydrocortisone in the treatment of SRNS. Moreover, glycyrrhizic acid alleviates osteoporosis induced by long-term use of glucocorticoids. These results indicate that Pm-GCH is a promising treatment strategy for SRNS.

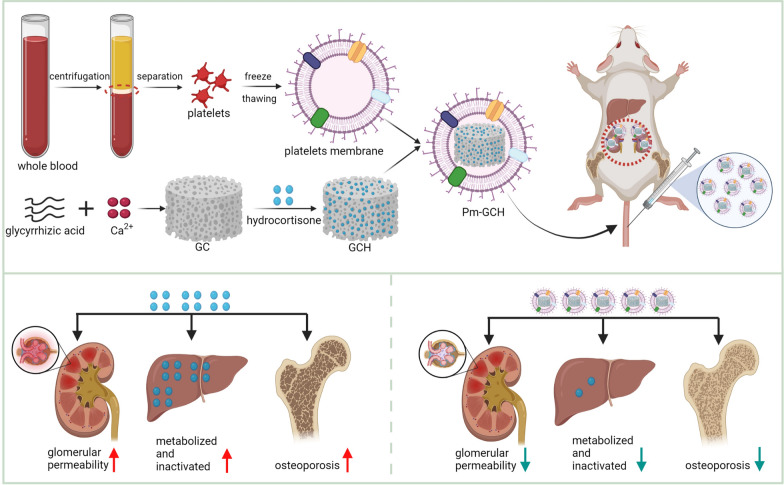

## Introduction

Primary nephrotic syndrome (PNS) is a clinical syndrome characterized by increased permeability of the glomerular filtration membrane and loss of a large amount of plasma protein from the blood, resulting in a high level of proteinuria, hypoproteinemia, hyperlipidemia and varying degrees of edema [1]. Most patients with PNS are sensitive to glucocorticoids, but 10–20% of patients do not have relieved proteinuria after 8 weeks of treatment with a sufficient amount of hormones, and thus develop steroid-resistant nephrotic syndrome (SRNS) [2]. The incidence of osteoporosis is up to 30% after long-term (over 6 months) use of high-dose glucocorticoids, and the incidence of glucocorticoid-induced osteoporosis (GIOP) ranks third in osteoporosis [3, 4]. Therefore, effective prevention and treatment of GIOP is very important in the treatment of SRNS. SRNS is a common disease caused by multiple factors. As a high-risk factor for nephrotic syndrome events, SRNS can induce renal tubulointerstitial fibrosis, which easily develops into end-stage renal disease and endangers the lives of patients [5, 6].

In vivo, glucocorticoids were mostly metabolized and inactivated by the liver microsomal cytochrome P450 enzyme system, resulting in low bioavailability. For example, hydrocortisone can be metabolized by P4503a (CYP3A) to 6β-hydroxy hydrocortisone, which is easily excreted from urine [7, 8]. Glycyrrhizic acid is a saponin component extracted from the traditional Chinese medicine licorice [9, 10]. Glycyrrhizic acid has anti-inflammatory, immunomodulatory, anticoagulant and steroid-like effects and is widely used in clinical practice [11, 12]. Studies have found that glycyrrhizic acid has an inhibitory effect on P450 enzymes, so it can be inferred that glycyrrhizic acid combined with hydrocortisone can reduce the inactivation of glucocorticoids in the liver and enhance its bioavailability [13]. Moreover, glycyrrhizic acid has steroid-like effects, and can be hydrolyzed to glycyrrhetinic acid, whose chemical structure is similar to that of steroid hormones, in vivo [14]. Glycyrrhetinic acid can bind to glucocorticoid receptors and has a weak glucocorticoid-like effect [15]. In addition, glycyrrhizic acid can block the production of inflammatory mediators by inhibiting the activity of phospholipase A2 and the classical activation pathway of complement C2, thus exerting anti-inflammatory effects [16, 17]. Glycyrrhizic acid has the structural characteristics of saponin amphiphilic substances and can interact with cholesterol or phospholipids on cell membrane to induce porosity and enhance membrane permeability. As a result, glycyrrhizic acid can freely pass through the membrane [18, 19]. Moreover, glycyrrhizic acid was shown to inhibit the differentiation of osteoclasts by regulating the NF-κB pathway, increase the mineral content of the femur, and have a protective effect on glucocorticoid-induced osteoporosis [20].

As a powerful driver in biomedical development, nanotechnology has shown great promise in the fields of early diagnosis, treatment, prevention of disease and bioengineering [21–23]. Nanomaterials loaded with traditional drugs can reduce the dosage of drugs and lead to targeted slow release [24]. Moreover, a multifunctional drug carrier system can be designed to achieve synergistic therapeutic effects [25]. Metal–organic frameworks (MOFs) are bridged by metal ions and organic ligands, which have a high specific surface area and porosity, have multiple ligand sites and show easy chemical modification [26, 27]. In this study, a porous metal–organic framework (GC) was prepared using glycyrrhizic acid and calcium ions (Ca^2+^).

Nanomaterials, as exogenous substances in the body, are easily eliminated [28]. To solve this problem, this study intends to use a natural platelet membrane to camouflage nanoparticles [29, 30]. The self-recognition molecule CD47 on the platelet membrane surface results in a prolonged cyclic half-life, enhanced immune escape, and incomparable biocompatibility of the core nanoparticles [31, 32]. In nephrotic syndrome, inflammation occurs in the kidney. Under the action of inflammatory factors, platelet membrane aggregates at the inflammatory site and plays a targeted role [33]. Furthermore, some studies have found that in the case of nephrotic syndrome, the abnormally activated platelets in the body aggregate at the inflammatory site, degranulate, release inflammatory factors and aggravate inflammation at the lesion site [34, 35]. Therefore, in this study, in addition to the targeting effect of platelet membrane vesicles, they could also compete with platelets in vivo to bind to inflammatory sites, reduce platelet activation and weaken the inflammatory response.

In this study, a metal–organic framework was constructed by glycyrrhizic acid and calcium ions with hydrocortisone (H) as the core, and a platelet membrane was used as the shell structure of the nanodrug system (Pm-GCH). Through platelet membrane camouflage, Pm-GCH not only nonspecifically targets the inflammatory site of nephrotic syndrome but can also compete with the inflammatory site and weaken the inflammatory response. In addition to a raw material for carrier synthesis, glycyrrhizic acid also has anti-inflammatory and inhibitory effects on glucocorticoid inactivation and anti-osteoporotic effects. Pm-GCH has a synergistic effect in the treatment of steroid-resistant nephrotic syndrome and provides a new therapeutic strategy for nephropathy.

## Materials and methods

### Materials

Glycyrrhizic acid (DG0006) was manufactured by Chengdu Desite Biological Technology Co., Ltd (Chengdu, China). Hydrocortisone (A610506) and calcium chloride (A501330) were purchased from Sangon Biotech Co., Ltd. (Shanghai, China). DMEM (11965092), RPMI 1640 (11875119), fetal bovine serum (10270106), penicillin streptomycin (15140122, 5000 U/ml), and trypsin (25200056) were purchased from Gibco (Thermo Fisher Scientific, USA). Serum-Free Cell Freezing Medium (C40100) was produced by New Cell & Molecular Biotech Co., Ltd. (Suzhou, China). The Calcein/PI Cell Viability/Cytotoxicity Assay Kit (C2015M) was produced by Beyotime Biotechnology (Shanghai, China). Millex Syringe Filters (400 nm and 200 nm) were purchased from Millipore (USA). The dialysis membrane (MW 1000D, YA1047) and hematoxylin–eosin Contamination Kit were purchased from SolarBio Science & Technology Co., Ltd. (Beijing, China). Anti-phosphoolipase A2 (ab239730) and Anti-component 2 (ab231651) monoclonal antibodies were manufactured by Abcam (USA). FITC-conjugated goat anti-mouse IgG (BA1101) and CY3-conjugated goat anti-rabbit IgG (BA1032) were obtained from Boster Biological Technology Co., Ltd. (Wuhan, China).

### Preparation of Pm-GCH

#### Preparation of platelet membrane vesicles (Pm)

As mentioned above, fresh mouse whole blood was placed in a sodium citrate anticoagulant centrifuge tube and centrifuged at 1000 rpm for 10 min, and the supernatant was collected to obtain platelet-rich plasma. Due to the presence of a certain amount of RBCs in the obtained platelet-rich plasma, 1% ammonium oxalate was added to lyse RBCs. After centrifugation at 3500 rpm for 15 min, the supernatant was discarded, and platelets were precipitated. Platelets were resuspended in normal saline, placed in a – 80 °C refrigerator for repeated freeze-thawing to release the platelets contents, and centrifuged at 5000 rpm for 15 min. The supernatant was discarded, and the membrane precipitate was washed repeatedly with PBS for 3 times and resuspended in water. Ultrasound (42 kHz, 100 W) was performed for 10 min. The particle size of Pm was detected by dynamic light scattering (DLS).

#### Construction of GCH

Glycyrrhitic acid (0.8 g) and calcium chloride (CaCl_2_, 0.1 g) were dissolved in 10 ml double distilled water, respectively. Glycyrrhitic acid was added dropwise to the CaCl_2_ solution and magnetically stirred until the solution turned milky-white to form GC. The solution was then centrifuged and washed with double distilled water 3 times, and the powder was lyophilized in a vacuum freeze dryer for later use. The morphological characteristics of GC were monitored by transmission electron microscopy (TEM) and scanning electron microscopy (SEM). PBS (2 ml) was used to dissolve GC (4 mg), and anhydrous ethanol (2 ml) was used to dissolve hydrocortisone (1.6 mg). GC and hydrocortisone (H) were mixed dropwise and stirred for 24 h, and then added to a dialysis bag to remove the unbound hydrocortisone to obtain GCH. The optical density (OD) of the dialysate at 235 nm was determined. The content, encapsulation efficiency (EE) and loading efficiency (LE) of hydrocortisone in the dialysate were calculated according to the following formula:

$${\text{EE}} = {\text{ mass of H into nanoparticles}}/{\text{mass of the feeding H}} \times {1}00\%$$

$${\text{LC}} = {\text{mass of H into nanoparticles}}/\left( {{\text{mass of GCs}} + {\text{mass of H into nanoparticles}}} \right) \times {1}00\%$$

#### The fusion of GCH and Pm

A constant volume (0.5 ml) of Pm (1.25 mg/ml) and GCH suspension (hydrocortisone level (H), 1 mg/ml) were mixed and then ultrasonicated in a water bath (30 min, 42 kHz, 200 W), followed by extraction through a 200 nm pore needle filter. After centrifugation at 5000 rpm for 15 min, the excess Pm in the supernatant was discarded, and the precipitate obtained was Pm-GCH.

#### Characterization of Pm-GCH

Zetasizer Nano (Malvern, UK) was used to detect the hydrodynamic size and zeta potential of Pm-GCH. TEM (HT7800, HITACHI, Japan) was used to detect the size and morphology of Pm-GCH. The UV–Vis absorption of GC (0.25 mg/ml), H (1 mg/ml), Pm (2 mg/ml) and Pm-GCH (1.5 mg/ml) was detected by a UV–Vis spectrometer (2600, Shimadzu, Japan). The platelet membrane protein composition was determined by SDS-PAGE. Then, Comassie Blue staining solution (P1305, SolarBio, China) was used to stain the gel. After decolorization, the gel imaging system was used for analysis.

### Release characteristics of glycyrrhizic acid and hydrocortisone in Pm-GCH in vitro

One milliliter of Pm-GCH was added to three dialysis bags and placed in PBS buffers at pH = 6.5 and pH = 7.4. Dialysate was taken at 2 h, 6 h, 12 h, 24 h, 48 h, and 72 h, and its absorbance at 235 nm (hydrocortisone characteristic absorption peak) and 252 nm (glycyrrhizic acid characteristic absorption peak) was detected. The absorbances of hydrocortisone and glycyrrhizic acid with different concentration gradients at 235 nm and 252 nm were determined, and the standard curves were established. The hydrocortisone and glycyrrhizic acid contents in the dialysate and drug release curve were calculated.

### Biocompatibility assessment in vitro

First, the blood compatibility of Pm-GCH was detected by a hemolysis test. Rabbit red blood cells (5%) were incubated with different concentrations of GCH and Pm-GCH in a water bath at 37 °C for 2 h. After centrifugation (1500 rpm, 10 min), the supernatant was collected, and its absorbance at 545 nm was measured using a UV–Vis spectrophotometer (Enspire 2300, PerkinElmer, USA). The immune evasive ability of Pm-GCH was assessed in a murine peritoneal macrophages (MPMs) uptake assay. The MPMs were incubated with Cy5-labeled GCH and Pm-GCH for 30 min and photographed under an inverted laser microscope (Zen2, Zeiss, Germany). Cell lysates were added to lyse the cells. The lysates were centrifuged and the supernatant was extracted. The fluorescence intensity at 664 nm was detected by a microplate detector (Enspire 2300, PerkinElmer, USA).

### Protective effect of Pm-GCH on adriamycin-injured 293T/D cells in vitro

First, the injury cell model of 293T cells (293T/D) treated with doxorubicin (6 μg/ml) was established. The 293T/D cells were divided into four groups, including the GC, H, GCH and Pm-GCH groups. 293T/D cells were treated with GC, H, GCH and Pm-GCH (H concentration 10 mg/L) for 24 h. A CCK8 cell viability test and staining of dead cells were carried out. CCK8 experiment: 293T/D cells were cultured in 96-well plates and then incubated with GC, H, GCH and Pm-GCH (H concentration 10 mg/L) for 24 h. Ten microliters of CCK solution was added to each well for 2 h. The absorbance at 450 nm was detected by a UV–Vis spectrophotometer. Staining test of living and dead cells: 293T/D cells were cultured in 6-well plate culture, and incubated with GC, H, GCH, and Pm-GCH (H concentration 10 mg/L) for further culture for 24 h. According to the instructions of Cytotoxicity Assay Kit (C2015M, Beyotime, China), 0.5 ml staining solution (Calcein-AM 2 μM, PI 4.5 μM) was added to each well and incubated at 37 °C for 5 min. The cells were observed and photographed under an inverted fluorescence microscope.

### Establishment of a mouse model of SRNS

Six-week-old female C57 mice were obtained from Hunan SJA Laboratory Animal Co., Ltd. All animal experiment schemes were approved by the Animal Use and Care Committee of Central South University. A mice model of SRNS was constructed according to the literature. A single injection of doxorubicin (6.2 mg/kg) was given through the tail vein, and hydrocortisone (25 mg/kg/day, 7 days) was injected into the hip muscle. Twenty-four-hour urine was collected on the 7th, 8th, and 9th days after injection for quantitative detection of urine protein. When the 24 h urine protein quantity was ≥ 100 mg/kg and lasted for more than 3 days, the model was considered to be successful.

### The distribution of Pm-GCH in vivo

Initially, 100 μl GCH-cy5 and Pm-GCH-cy5 (H, 1 mg/ml) were injected through the tail vein of SRNS mice. The SRNS mice were anesthetized at 6, 24, and 48 h after injection and imaged with the In Vivo Imaging System (PerkinElmer, USA). Meanwhile, mice were euthanized at 6, 24 and 48 h after injection. The heart, liver, spleen, lung, kidney and brain were collected for fluorescence imaging by an In Vivo Imaging System (PerkinElmer, USA).

### The protective effect of Pm-GCH in vivo

Then, 100 μl GC, H, GCH and Pm-GCH (H, 1 mg/ml) were injected daily through the tail vein of SRNS mice for 3 consecutive days. Urine was collected for 24-h urinary protein quantitation. The concentration of urine protein was detected with a BCA Protein Assay Kit (P0012S, Beyotime, China). After 12 days, all SRNS mice were euthanized, and fresh whole blood was taken for routine blood and kidney function. Hematological indices (RBCs, Hb, WBCs, PLTs) were determined by an automatic blood cell analyzer (Mindray, China). The liver function index (ALT, AST) and renal function index (CREA, BUN) were detected by a Leadman Diagnostic Kit (Leadmanbio, China). Kidney, lung, liver, spleen, heart and femur samples were fixed with 4% paraformaldehyde and embedded in paraffin. Ultrathin sections were used for HE and Masson staining with a hematoxylin–eosin staining Kit (G1120, Solarbio, China) and Masson’s trichrome stain kit (G1340, Solarbio, China).

### Transmission electron microscope imaging

The kidney tissues (< 1 mm^3^) were fixed in 2.5% glutaraldehyde at 4 °C overnight, and then fixed in 1% osmic acid for 1–2 h. The samples were dehydrated with gradient concentrations of ethanol. Then, kidney samples were treated with acetone for 20 min and embedded with an Embed 812 Embedding Kit (GE14120, EMS, USA). The samples were sliced with an ultra-thin slicer to obtain sections of 70–90 nm. The sections were stained with uranyl acetate and alkaline lead citrate and observed using transmission electron microscopy (Hitachi, Japan).

### Immunofluorescent staining

Paraffin sections were dewaxed with xylene and hydrated with different gradients of alcohol. Sections were prepared in citrate buffer at 95 °C for antigen repair. Hydrogen peroxide (3%) was added to the sections to remove endogenous peroxidase activity, and 5% BSA was added to block the nonspecific binding site. The sections were incubated with PLA2 primary antibody (1:200) at 37 °C for 2 h. After washing with PBS, FITC-labeled goat anti-mouse secondary antibody (1:500) was added on the sections at 37 °C for 2 h. After washing with PBS, the sections were incubated with C2 primary antibody (1:200) and CY3-labeled goat anti-rabbit secondary antibody (1:500) at 37 °C for 2 h. Finally, kidney sections were restained with DAPI and photographed with a fluorescence microscope.

### Micro-CT analysis

After the application of Pm-GCH, the femurs of mice were taken for micro-CT scanning (SkyScan1276, Bruker, Germany). The following parameters were obtained and analyzed: BV, bone volume; TV, tissue volume; BV/TV, relative bone volume; Tb. Th, trabecular thickness; and Tb. N, trabecular number.

### Statistical analysis

SPSS software 18.0 was used for statistical analysis. Data were presented as the mean ± standard deviation. Comparisons between groups were analyzed with one-way analysis of variance (ANOVA) and Tukey’s post test. One asterisk (*) represents p < 0.05, and a double asterisk (**) represents p < 0.01.

## Results and discussion

### Construction and characterization of Pm-GCH

**Fig. 1:**
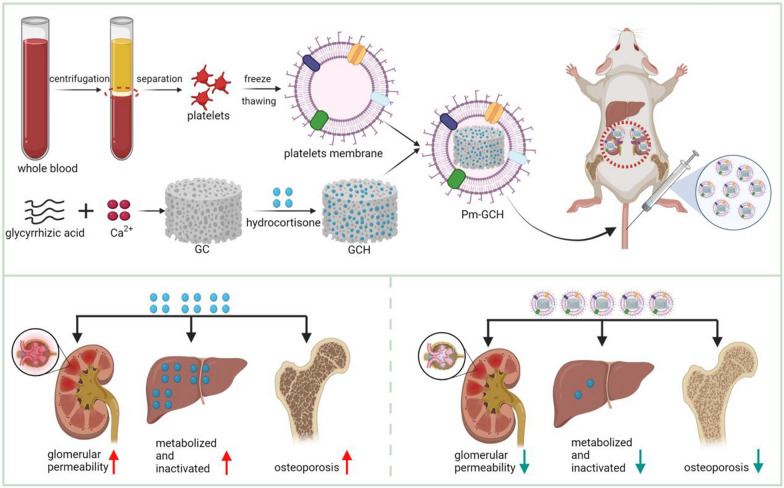
Construction of Pm-GCH and its targeted therapeutic mechanism in SRNS by inhibitting inflammation and osteoporosis

**Fig. 2:**
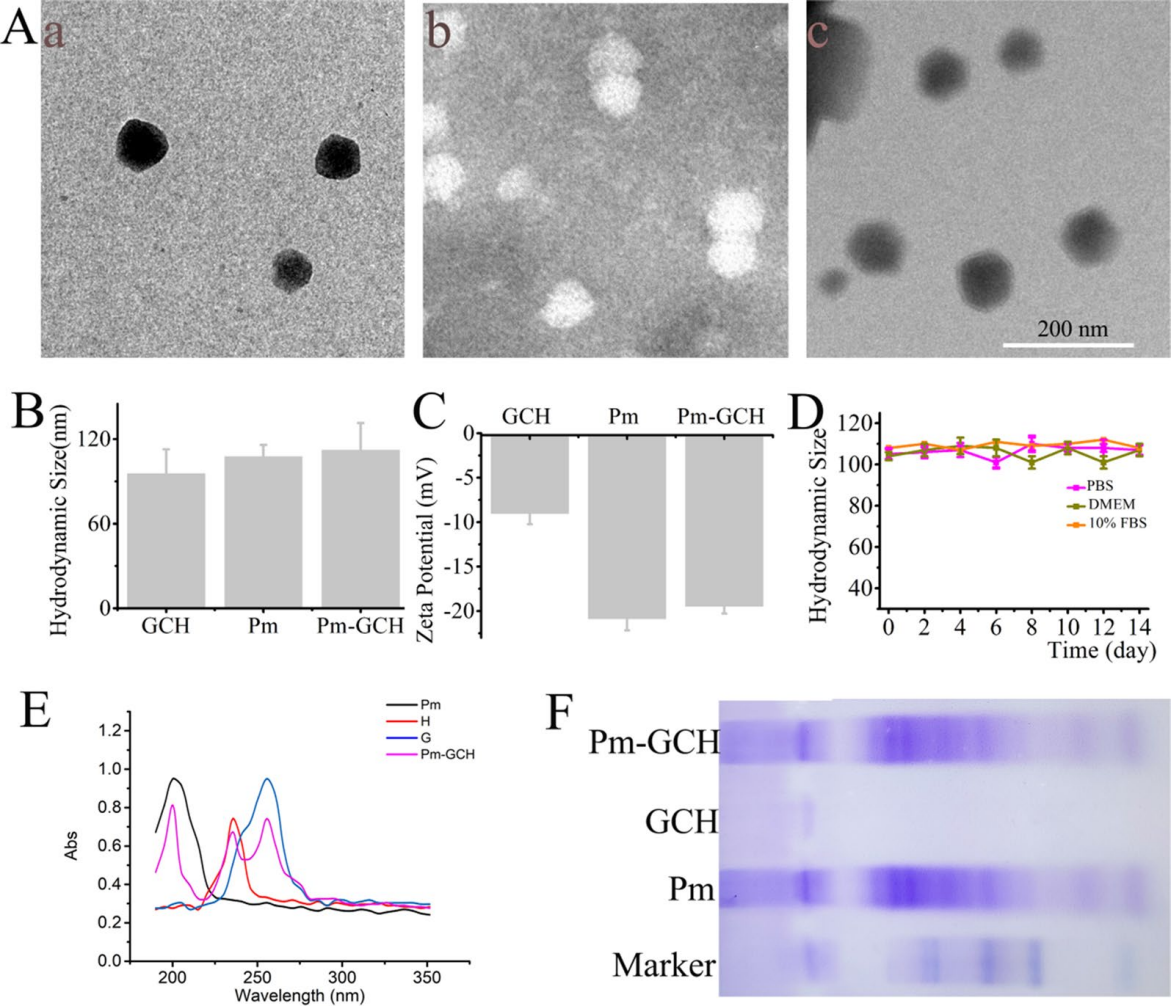
Characterization of Pm-GCH. **A** TEM-image analysis of GCH (a), Pm vesicles (b), and Pm-GCH (c). Scale bar: 200 nm. **B** Hydrodynamic size and **C** zeta potential of GCH, Pm and Pm-GCH. **D** The size change of Pm-GCH over 14 days in PBS, DMEM and 10% FBS. **E** UV–vis spectra of Pm, H, G and Pm-GCH. **F** The profile of platelet membrane proteins was detected by SDS-PAGE analysis

As shown in Fig. 1, the preparation process of Pm-GCH mainly includes the generation of a metal–organic framework (GC) constructed by glycyrrhizic acid and Ca^2+^ loading hydrocortisone to form a GCH nanocore. The shell structure, platelet membrane vesicle (Pm), wrapped GCH to form the final core–shell structure of Pm-GCH. First, the changes in the size, zeta potential, and morphology of Pm-GCH were determined. In Fig. 2A, the TEM image indicates that Pm-GCH had a typical core–shell structure. The particle size of GCH was approximately 95 ± 5 nm. After fusion with Pm, the particle size of Pm-GCH was 110 ± 9 nm. In Fig. 2B, the dynamic light scattering data show that the particle sizes of GCH, Pm, and Pm-GCH are 98 ± 13 nm, 102 ± 9 nm and 121 ± 12 nm, respectively. In Fig. 2C, the zeta potentials of GCH and Pm were − 9 ± 1.2 mV and − 21 ± 1.6 mV, respectively, while the zeta potential of Pm-GCH was − 19 ± 0.9 mV, close to the zeta potential of Pm, which may be due to the charge shielding effect caused by Pm encapsulation, and demonstrates the successful encapsulation of Pm-GCH. To assess the dispersion and stability of Pm-GCH, we monitored the hydrodynamic size of Pm-GCH for two weeks in PBS, DMEM and 10% FBS, and there was no significant change in particle size. The above results indicated that Pm-GCH possessed good stability in physiological solution (Fig. 2D). In the UV–Vis absorption spectrum (Fig. 2E), Pm-GCH had absorption peaks at 200 nm, 235 nm and 252 nm, respectively, in accordance with the characteristic absorption peaks of Pm, hydrocortisone and glycyrrhizic acid, respectively. In the SDS-PAGE electrophoresis image, the protein profile of Pm-GCH was similar to that of Pm, which confirmed once again that the Pm protein was successfully transferred to Pm-GCH (Fig. 2F).

### Hemocompatibility and immune escape effect of Pm-GCH

**Fig. 3:**
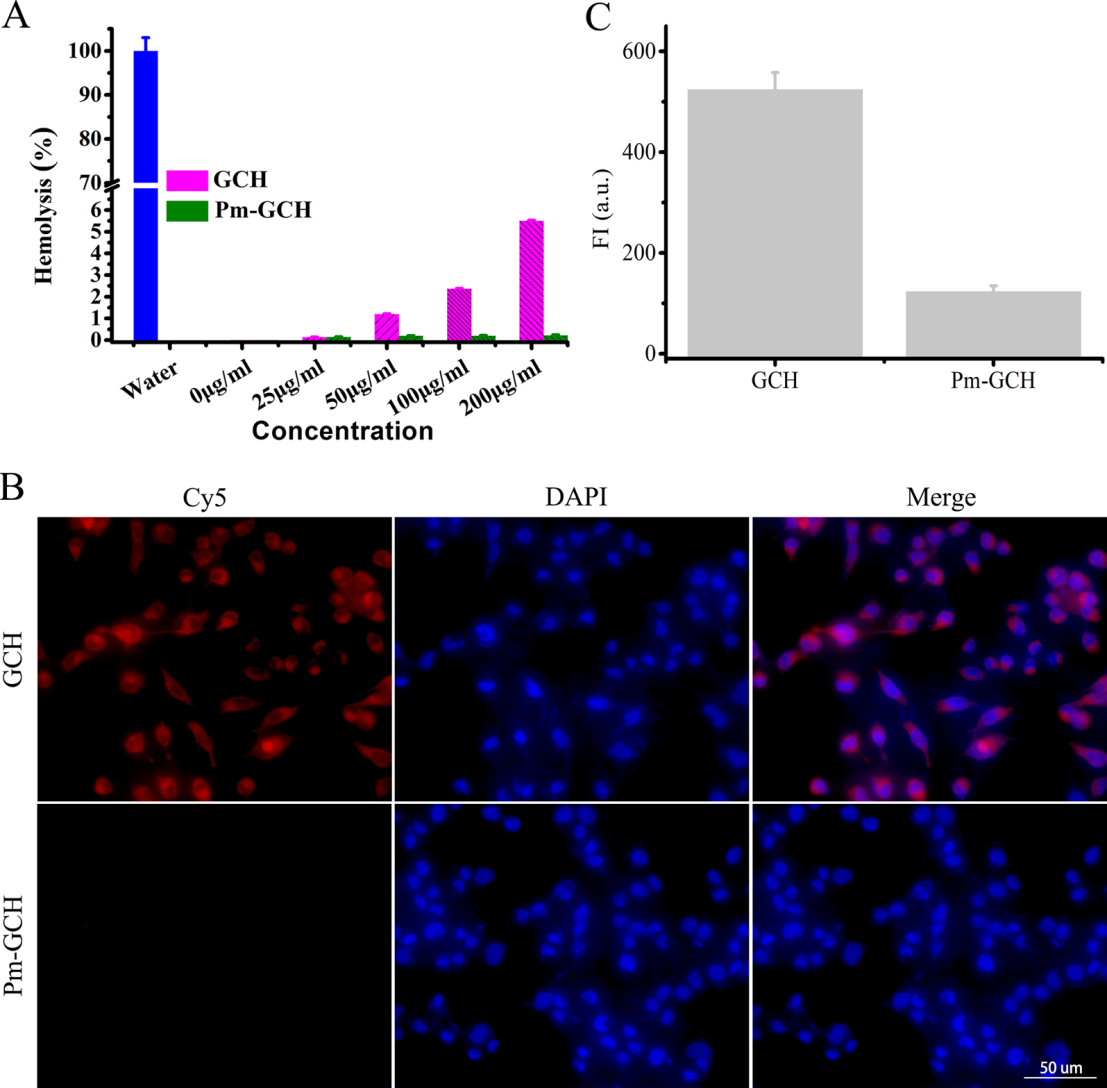
The hemocompatibility and immune escape effect of Pm-GCH. **A** The hemolysis rate of RBCs with different concentrations of GCH and Pm-GCH. **B** The fluorescence images of macrophages aftter treated with GCH and Pm-GCH for 6 h. **C** The fluorescence intensity of macrophage lysates after treated with GCH-Cy5 and Pm-GCH-Cy5 for 6 h

First, the hemocompatibility of Pm-GCH was evaluated by a hemolysis test. Different concentrations of GCH and Pm-GCH were added to 5% RBCs for 2 h. After centrifugation, the absorbance of the supernatant was detected. As indicated in Fig. 3A, the hemolysis rate of GCH (200 μg/ml) was approximately 5.4%, while the hemolysis rate of Pm-GCH at various concentrations was less than 1%. This finding suggests that Pm-GCH has good blood compatibility after being encapsulated by the platelet membrane and can be used for intravenous administration.

Second, the immune evasive ability of Pm-GCH-Cy5 was investigated by detecting the antiphagocytic effect on macrophages. As shown in Fig. 3B and C, a large amount of Cy5 (red fluorescence) was present in macrophages in the GCH-treated group. In contrast, the red fluorescence in the Pm-GCH-treated macrophages was significantly reduced, indicating that the effect of macrophages on the phagocytosis of Pm-GCH was significantly reduced after platelet membrane vesicle camouflage. The above results confirm that GCH with platelet membrane encapsulation can evade the identification of macrophages and reduce the clearance of the immune system.

### The drug loading and release of Pm-GCH

**Fig. 4:**
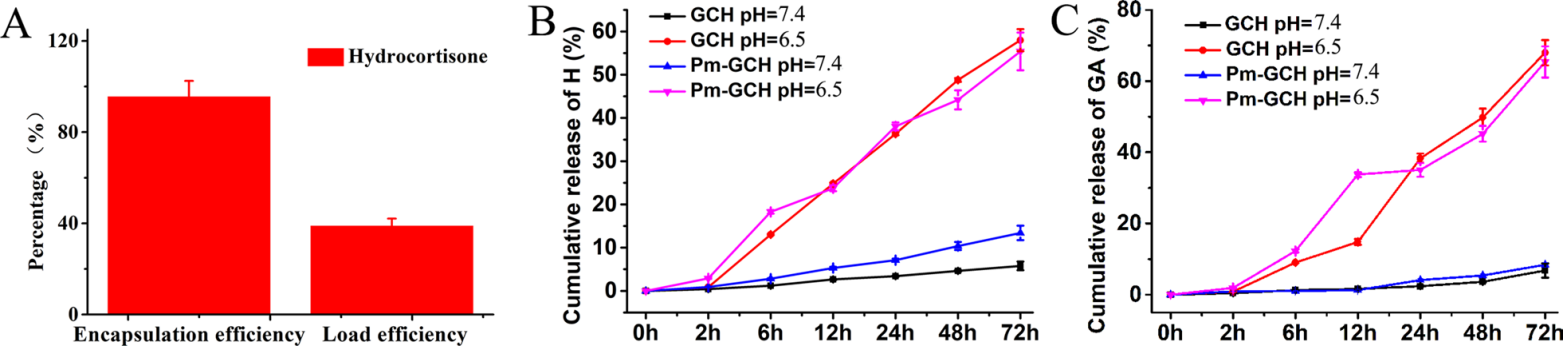
**A** The EE and LE of hydrocortisone. **B** Cumulative release ratio of H from GCH and Pm-GCH at different pH values (6.5, 7.4). **C** Cumulative release ratio of GA from GCH and Pm-GCH at different pH values (6.5, 7.4)

As a metal–organic material, GC prepared with glycyrrhizin and Ca^2+^ as raw materials has high specific surface area and can serve as an ideal carrier for drugs. As indicated in Fig. 4A, the EE and LE of hydrocortisone load on GC are 96.7% and 38.7%, respectively. Furthermore, an ideal drug carrier should also achieve efficient drug release at the targeted site. During this research, pH 7.4 and pH 6.5 simulated a neutral circulation environment and an acidic inflammatory microenvironment, respectively. As indicated in Fig. 4B, C, Pm-GCH released hydrocortisone and glycyrrhizic acid in a pH-dependent manner. Compared with results at pH 7.4, hydrocortisone and glycyrrhizic acid were released more easily at pH 6.5, which demonstrates that Pm-GCH is particularly useful for drug delivery under renal inflammation conditions in steroid-resistant nephrotic syndrome.

### Biocompatibility and protective effect of Pm-GCH in vitro

**Fig. 5:**
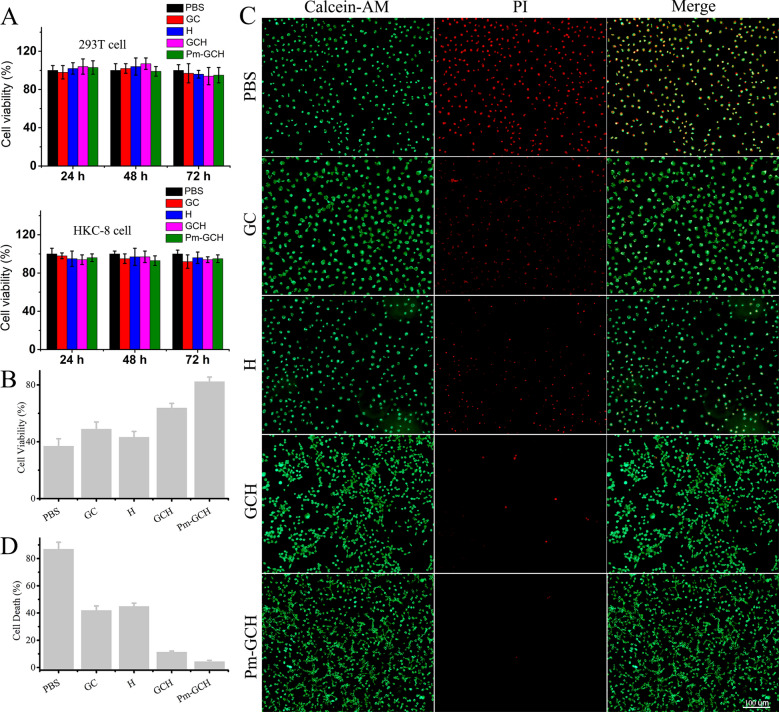
**A** The viability of 293T and HKC-8 cells was detected by CCK8 assays after administration of PBS, GC, H, GCH and Pm-GCH for 24, 48 and 72 h. **B** The viability of 293T/D cells was detected by CCK8 assays after administration of PBS, GC, H, GCH and Pm-GCH for 24 h. **C** Live/dead staining of 293T/D cells after various treatments (PBS, GC, H, GCH and Pm-GCH) for 24 h. **D** Semiquantitative ratio of cell death

First, the biocompatibility of Pm-GCH in vitro was assessed in 293T and HKC-8 cells. Cells were treated with GC, H, GCH, and Pm-GCH for 24, 48, and 72 h at 37 °C. As shown in Fig. 5A, the viability of 293T and HKC-8 cells incubated with Pm-GCH for 72 h was 95.5 and 94.3%, respectively. The CCK8 assay indicated that Pm-GCH showed no significant cytotoxicity and had good biocompatibility. To explore the protective effect of Pm-GCH in vitro, an injuried 293T cell model (293T/D) treated with doxorubicin (6 μg/ml) was established. The 293T/D cells were divided into four groups: the GC, H, GCH and Pm-GCH groups. As shown in Fig. 5B, in the CCK-8 assays, GC, H, GCH, and Pm-GCH all enhanced 293T/D cell viability to varying degrees. At the same time, Pm-GCH exerted the strongest cell protective effect. The protective effect of Pm-GCH in vitro was evaluated by living/dead cell staining. After 293T/D cells were treated with GC, H, GCH, and Pm-GCH, cells were stained with calcein-AM/PI staining solution. The living cells showed green fluorescence with calcein-AM staining, and the dead cells showed red fluorescence with PI staining. The staining results of living and dead cells were consistent with those of the CCK-8 assay (Fig. 5C, D). After Pm-GCH treatment, the number of apoptotic/dead cells with red fluorescence was significantly reduced, which indicated that Pm-GCH showed the best protective effect in vitro. It was reported that glycyrrhizic acid ameliorated cisplatin-induced nephrotoxicity by inhibiting the cytoplasmic localization and release of HMGB1 [36]. Glycyrrhizic acid was aslo found to have a protective effect on renal tubular epithelial cell proliferation and oxidative stress induced by high glucose by increasing the expression of AMPK, SIRT1, and Mn-SOD [37]. In addition, hydrocortisone could inhibit doxorubicin-induced cardiomyocyte apoptosis by promoting the expression of pro-survival protein Bcl-XL [38]. In conclusion, the protective effect of Pm-GCH on renal epithelial cells lies in the nonspecific targeting effect of Pm on injured cells and the combined application of glycyrrhizic acid and hydrocortisone.

### Biodistribution of Pm-GCH in vivo

**Fig. 6:**
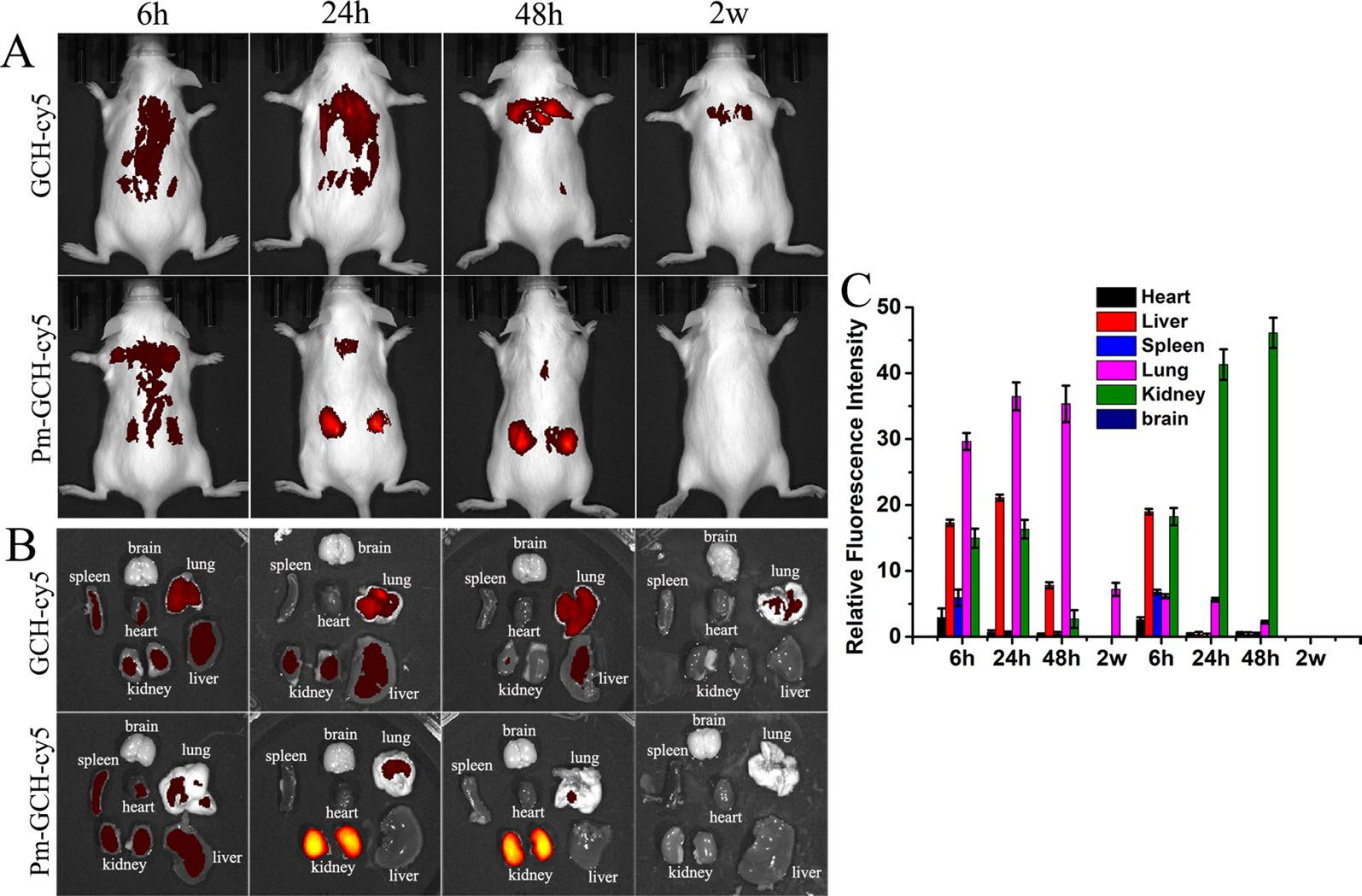
In vivo targeting potential of Pm-GCH. **A** The biodistribution images of the whole body of mice. **B** Fluorescence images of main organs (brain, heart, liver, spleen, lung, and kidney) were measured at 6 h, 24 h, 48 h and 2 week after intravenous injection of GCH and Pm-GCH in SRNS mice. **B** Semiquantitative assessment of fluorescent signals in brain, heart, liver, spleen, lung, and kidney

First, a mouse model of SRNS was constructed. When the 24 h urine protein was ≥ 100 mg/kg and lasted for more than 3 days, the model was considered to be successful. Then, 100 μl of PBS, GCH-Cy5 and Pm-GCH-Cy5 was injected daily through the tail vein. Six to 48 h after injection, the SRNS mice were imaged by an IVIS^®^ Spectrum In Vivo Imaging System (Fig. 6A). Meanwhile, the SRNS mice were euthanized, and the kidney, heart, liver, spleen and lung were collected and imaged by an IVIS^®^ Spectrum In Vivo Imaging System (Fig. 6B, C). As shown in Fig. 6, less GCH-Cy5 reached the inflammation site of the kidney from 6 to 48 h. Because of the targeting of inflammation and immune escape, the accumulation of Pm-GCH-Cy5 in the inflammation site of the kidney increased significantly at 24 h. Meanwhile, GCH-Cy5 showed less accumulation in the kidney and was mainly concentrated in the lung and liver. In contrast to GCH-Cy5, the content of Pm-GCH-Cy5 in the lung and liver was obviously lower. Notably, Pm-GCH-Cy5 showed greater accumulation than GCH-Cy5 in the kidney. Two weeks after injection, there was no Pm-GCH and still a small amount of GCH distributed in the mice, which is mainly because that GCH tended to accumulate in the body and blocked the pulmonary vessels. The above results suggest that the immune escape of Pm-GCH-Cy5 prolongs the blood circulation time and achieves targeted aggregation in kidney sites under the effect of Pm.

### The renal protection of Pm-GCH in vivo

**Fig. 7:**
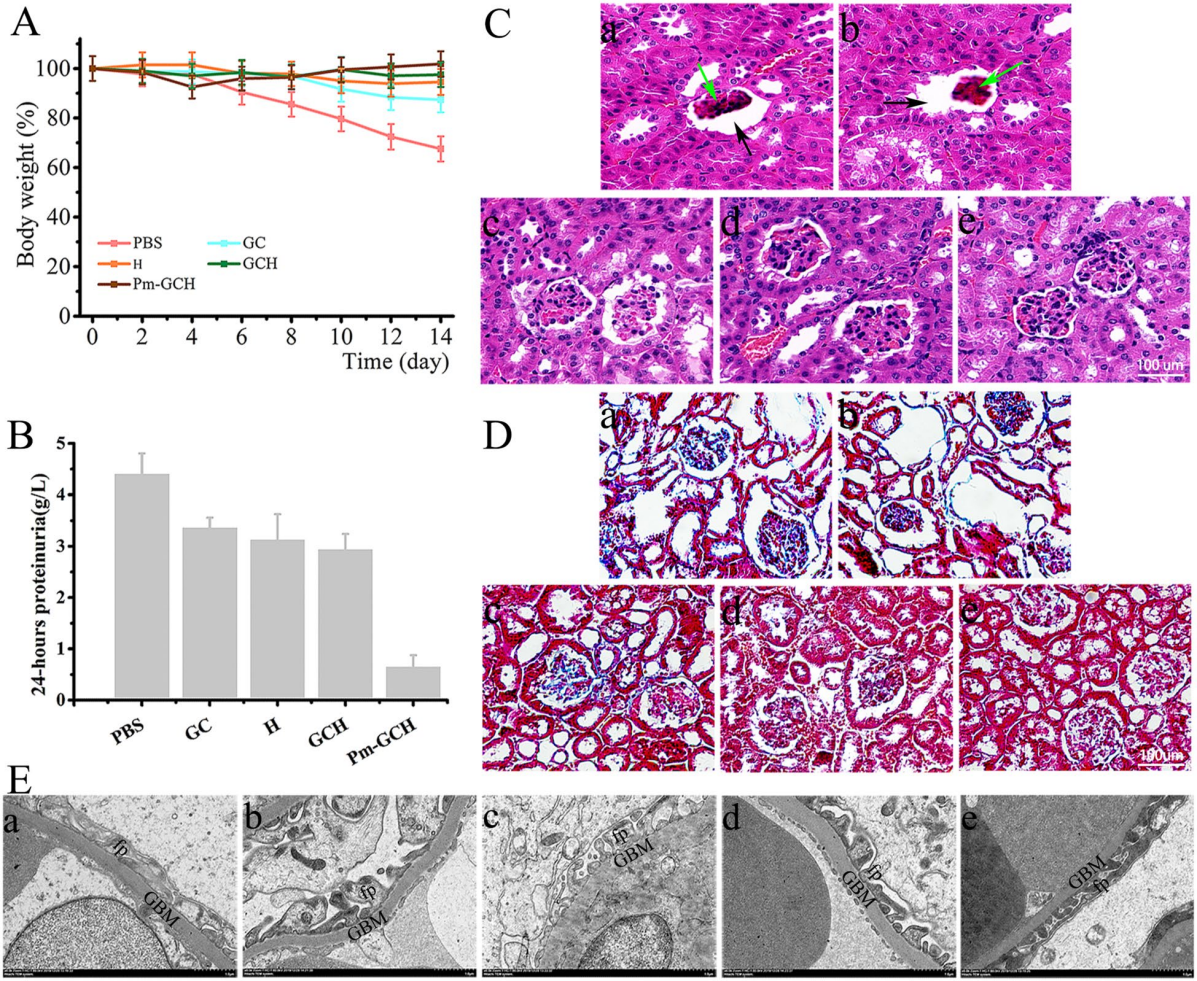
**A** Body weight alterations in the mice with SRNS during GC, H, GCH and Pm-GCH treatment. **B** Quantification of 24-h proteinuria after GC, H, GCH and Pm-GCH treatment. Representative images of kidneys after H&E staining (**C**) and Masson staining (**D**) after intravenous injection of (a) PBS, (b) GC, (c) H, (d) GCH and (e) Pm-GCH, respectively. Black arrows, enlarged Bowman’s space; green arrows, glomerular sclerosis. **E** The ultrastructure of the glomerular filtration barrier was assessed by transmission electron microscopy. *fp* foot process, *GBM* glomerular basement membrane

**Fig. 8:**
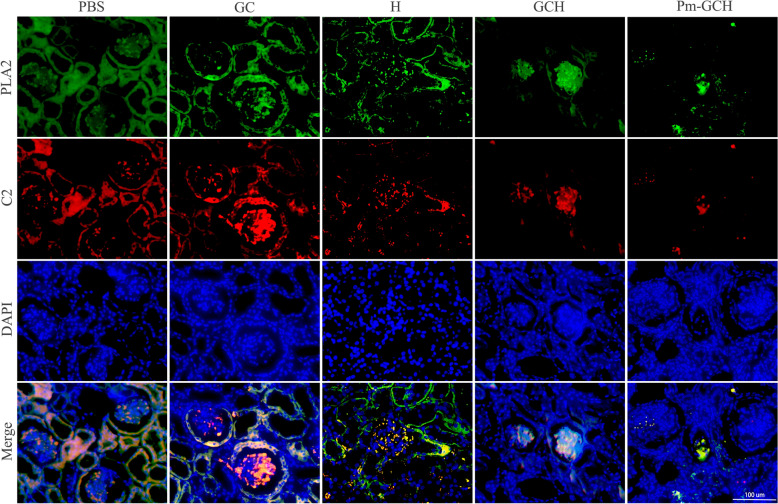
Expression pattern of inflammatory cytokines (PLA2 and C2) analyzed by immunofluorescence staining in the kidney

Next, the renal protective effects of Pm-GCH in vivo in the SRNS mice were evaluated. When the 24 h urine protein quantity was ≥ 100 mg/kg and lasted for more than 3 days, the model was considered to be successful. PBS, GC, H, GCH and Pm-GCH (100 μl) were administered through the tail vein. The body weight and 24 h urine protein of the mice with SRNS were monitored continuously within 14 days (Fig. 7A, B). The mice with SRNS showed greater albuminuria and weight loss. GC, H, GCH and Pm-GCH all reduced albuminuria and improved body weight to some extent. Notably, Pm-GCH had the most significant effect. Furthermore, the results of HE and Masson staining showed glomerular sclerosis, enlarged Bowman’s space and increased collagen deposition in the kidneys of the mice with SRNS (Fig. 7C, D). In the Pm-GCH group, glomerular sclerosis and collagen deposition were significantly improved. In addition, the ultrastructure of the glomerular filtration barrier was observed by transmission electron microscopy. As shown in Fig. 7E, podocyte foot process (fp) effacement was observed in the mice with SRNS, resulting in fusion of the podocyte foot process, loss of slit diaphragm, and microvillous transformation. Interestingly, Pm-GCH reproduced the foot process of the podocyte and slit diaphragm. The pathogenesis of nephrotic syndrome is complex, and inflammation has been proven to be an important cause. During nephrotic syndrome, the abnormal expression of inflammation-related protein PLA2 and complement C2 activates the inflammatory response and aggravates renal function injury [39]. PLA2 can breakdown phospholipids to produce arachidonic acid, which is further metabolized to produce cyclooxygenase and prostaglandin E2, inducing an inflammatory response and being the trigger point of the inflammatory response [40]. Complement C2 is the rate-limiting component of the complement activation cascade [41]. To study the anti-inflammatory mechanism of Pm-GCH, we detected inflammatory cytokines (PLA2, C2) in the kidneys by immunofluorescence staining. As shown in Fig. 8, in the kidneys of the mice with SRNS, inflammatory cytokines (PLA2, C2) were highly expressed. In the Pm-GCH group, the levels of inflammatory cytokines (PLA2 and C2) were downregulated. The above results suggested that Pm-GCH improved the inflammatory response by inhibiting the activity of PLA2 and C2.

### The anti-osteolytic effect of Pm-GCH in vivo

**Fig. 9:**
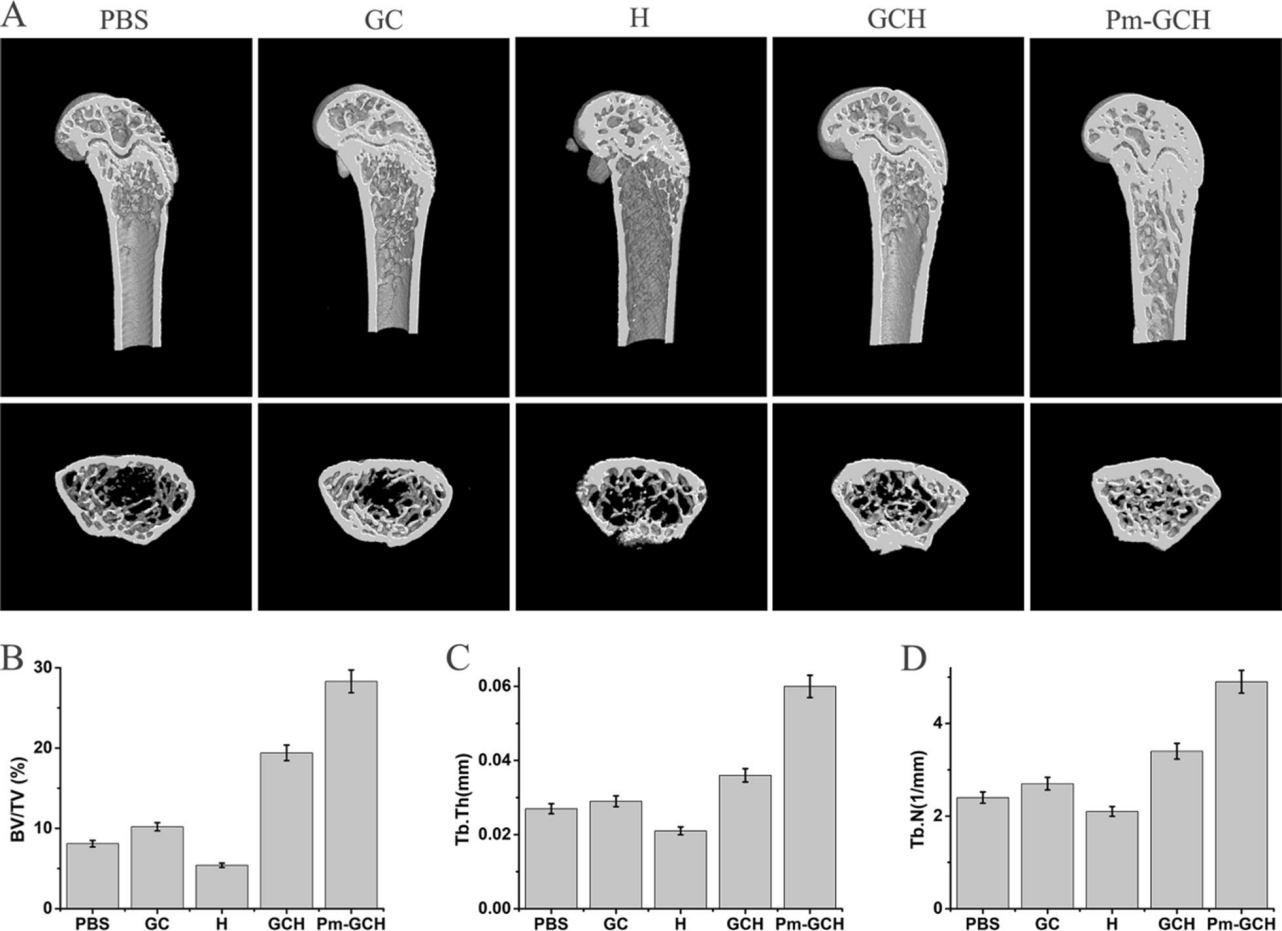
Effect of Pm-GCH on the femur of the mice with SRNS. **A** Three-dimensional reconstruction images of femurs obtained by micro-CT. Bone parameters including BV/TV (**B**), Tb. Th (**C**) and Tb. N (**D**) were calculated

Large amounts of glucocorticoids were used in the modeling of the mice with SRNS. Long-term use of high doses of glucocorticoids leads to osteoporosis. It was reported that glycyrrhizic acid promoted osteogenic differentiation of bone marrow stromal cells by activating the Wnt/β-catenin signaling pathway [42]. In addition, as an 11β-hydroxysteroid dehydrogenase inhibitor, glycyrrhizic acid has a protective effect on glucocorticoid-induced osteoporosis [43]. To investigate whether Pm-GCH improves glucocorticoid-induced osteolysis, we performed CT imaging of the mice femurs (Fig. 9A). Micro-CT 3D reconstructions indicated a decrease in the number of femoral trabeculae and the thickness of the femoral shaft in the mice with SRNS. However, as shown in Fig. 9B, the mice treated with Pm-GCH had significantly higher trabecular bone volume per total volume (BV/TV) values than the mice with SRNS. Furthermore, trabecular thickness (Tb. Th) and trabecular number (Tb. N) in the Pm-GCH-treated mice were significantly increased (Fig. 9C, D). These results indicated that PM-GCH significantly improved osteolysis induced by glucocorticoids.

### The biocompatibility of Pm-GCH in vivo

**Fig. 10:**
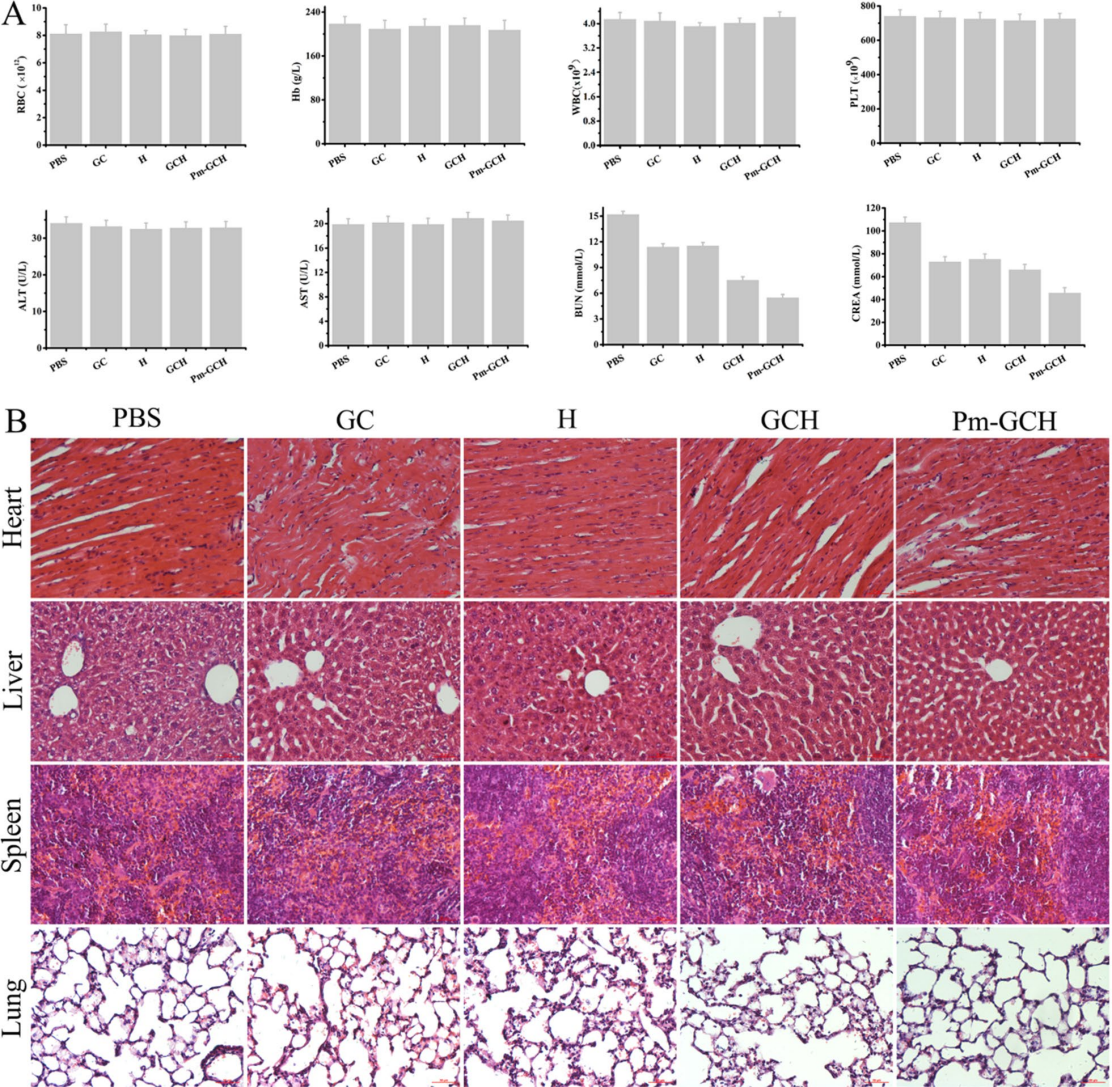
**A** Hematological and hematobiochemical analysis of peripheral blood from the mice with SRNS administrated with PBS, GC, H, GCH and Pm-GCH. (a) RBC, (b) Hb, (c) WBC, (d) PLT, (e) ALT, (f) AST, (g) BUN and (h) CREA. **B** HE staining images of mice heart, liver, spleen and lung slices from different treatment groups at the 14th day after different treatments

The biocompatibility of Pm-GCH was also evaluated in vivo. As shown in Fig. 10A, there were no obvious differences in the hematological index (RBCs, Hb, WBCs, PLTs) or liver function index (ALT, AST) after GC, H, GCH and Pm-GCH treatment. However, Pm-GCH improved the elevation of the renal function index (CREA, BUN) induced by SRNS. Moreover, HE staining of the heart, liver, spleen and lung showed that Pm-GCH did not change the histological structures of major organs (Fig. 10B). These results further verify that Pm-GCH possesses good biocompatibility.

## Conclusion

In this study, MOFs were constructed by glycyrrhizic acid and Ca^2+^, loaded with hydrocortisone, and platelet membrane vesicles were used for camouflage to target the inflammatory sites of the kidney in an SRNS model. Glycyrrhizic acid plays an anti-inflammatory role, an immunomodulatory role, and a steroid-like role, enhances the effect of glucocorticoids and reduces the side effects of glucocorticoid application. Glucocorticoids interfere with arachidonic acid metabolism, inhibit the synthesis of cytokines and rapidly inhibit inflammation and the immune response to improve glomerular membrane permeability, diuresis and eliminate urinary protein. Nanoscale platelet membrane vesicles compete with platelets in vivo to bind to inflammatory sites, reduce platelet activation and weaken the inflammatory response. The application of this multifunctional targeted bionic sustained-release nanomedicine system can reduce the dose of glucocorticoids, improve glucocorticoid resistance and osteolysis, and enhance the therapeutic effect of nephrotic syndrome through the synergism of glycyrrhizin, glucocorticoids and platelet membrane vesicles.

## Data Availability

All data generated or analyzed during this study are included in this published article.
